# Does Physically Demanding Work Hinder a Physically Active Lifestyle in Low Socioeconomic Workers? A Compositional Data Analysis Based on Accelerometer Data

**DOI:** 10.3390/ijerph15071306

**Published:** 2018-06-21

**Authors:** Charlotte Lund Rasmussen, Javier Palarea-Albaladejo, Adrian Bauman, Nidhi Gupta, Kirsten Nabe-Nielsen, Marie Birk Jørgensen, Andreas Holtermann

**Affiliations:** 1 National Research Centre for the Working Environment, 2100 Copenhagen, Denmark; ngu@nfa.dk (N.G.); nabe@sund.ku.dk (K.N.-N.); aho@nfa.dk (A.H.); 2 Department of Public Health, Section of Social Medicine, University of Copenhagen, 2100 Copenhagen, Denmark; 3 Biomathematics and Statistics Scotland, Edinburgh EH9 3FD, UK; javier.palarea@bioss.ac.uk; 4 Prevention Research Collaboration, School of Public Health, University of Sydney, Sydney 2006, Australia; adrian.bauman@sydney.edu.au; 5 Department of Forensic Science, University of Copenhagen, 2100 Copenhagen, Denmark; marie.birk.joergensen@sund.ku.dk; 6 Department of Sports Science and Clinical Biomechanics, University of Southern Denmark, 5230 Odense, Denmark

**Keywords:** physical activity, leisure time, blue-collar, low status occupation, work-life balance, time-use epidemiology

## Abstract

Leisure time physical activity (LTPA) is strongly associated with socioeconomic position (SEP). Few studies have investigated if demanding occupational physical activity (OPA) could impede a physically active lifestyle in low SEP groups. The aim of this study was to investigate the association between OPA and LTPA among low SEP men and women. We used cross-sectional data from 895 low SEP workers who wore accelerometers for 1–5 consecutive workdays. The associations between the relative importance of activities performed during work and leisure time were assessed using compositional regression models stratified on sex. Compositional isotemporal substitution models were used to assess the implication of increasing occupational walking, standing, or sitting on LTPA. We found dissimilarity in LTPA between the sexes, with men spending more waking leisure time sedentary than women (men ~67%, women ~61%), suggesting women performed more household tasks. In men, the associations between OPA and LTPA were weak. In women, the strongest association was observed between the relative importance of occupational walking and leisure time standing ($\hat{\beta }$ = −0.16; *p* = 0.01), where reallocating 15 min work time to occupational walking showed an expected decrease in leisure time standing of 7 min. If this time was spent on additional sedentary leisure time, it could have adverse health consequences.

## 1. Introduction

Leisure time physical activity (LTPA) is a strong protective factor for several non-communicable diseases and all-cause mortality [1,2,3]. However, LTPA is also socially disproportionally distributed: the lower the socioeconomic position (SEP), the worse the health and likelihood of engaging in LTPA [4,5,6]. Accordingly, public health strategies aim to increase LTPA in this high-risk population [7,8]. To succeed, modifiable determinants of LTPA among low SEP groups need to be identified.

Several individual determinants of LTPA among low SEP groups are well established, including age, sex and overall health status [9,10]. By contrast, structural factors are the least understood but an important class of determinants in low SEP groups by not relying on individual knowledge, motivation or resources [11,12]. One modifiable structural factor suggested as a barrier for LTPA among low SEP groups is demanding occupational physical activities (OPA), being highly prevalent in this group [11].

Low position occupations often include job tasks requiring stationary and monotonous behaviors, such as stationary standing and extensive walking [13,14]. Stationary standing requires sustained muscle contraction, causing increased intramuscular pressure periods and thereby decreasing blood flow to the muscles [15]. Consequently, the muscles receive less oxygen, leading to muscle fatigue which can persist up to 24 h after the end of the work period [15,16]. Furthermore, prolonged exposure to working whilst upright, i.e., walking or stepping, has been found to induce pain in lower extremities [14,17]. Accordingly, high levels of OPA could exhaust workers, leaving little energy for being physically active during leisure time. Furthermore, workers with manual jobs might believe that they obtain the recommended level of daily physical activity during work hours and thus, lack motivation to engage in LTPA [18].

The potential adverse influence of OPA on LTPA is understudied and unclear [11]. Studies report that individuals with high levels of OPA compensate by spending less leisure time on physical activities [19,20,21]. Other studies find individuals with physically active jobs being more likely to perform physical activity during leisure time [22,23,24]. These discrepancies could be caused by methodological inconsistency. Some studies have used self-reported information on physical activity levels [19,20,22,23], which are prone to issues of recall- and response-bias [25]. Accordingly, the use of technical measurements for assessing OPA and LTPA has been recommended [25,26]. Another reason for the inconsistent findings could be differences in study populations. The majority of studies are based on samples strongly dominated by middle or higher SEP groups [19,21,22,23,24,27]. However, having lower SEP groups underrepresented in study samples hinders inferences on the effect of OPA and LTPA in this high-risk population.

Finally, no study investigating the unfavorable influence of OPA on LTPA among low SEP individuals has taken the full 24 h day into account. Time spent on physical activities and sleep are exhaustive parts of a finite whole (24 h day) [28,29]. Hence, the portions of time spent on each activity throughout the day represent relative information and are co-dependent and collinear [30]. The statistical analysis of such constrained data (known as compositional data) requires a special methodology; log-ratio analysis [30,31,32]. Accordingly, the aim of this study was to investigate the association between OPA and LTPA in a group of low SEP men and women, using compositional data analysis.

## 2. Materials and Methods

This was a cross-sectional study based on baseline data from two Danish studies: the Danish PHysical ACTivity cohort with Objective measurements (DPhacto) [33] and the New Method for Objective Measurements of Physical Activity in Daily Living (NOMAD) study [34]. DPhacto and NOMAD were identical in data procedures and collection, which facilitated merging the data. Details of the studies have been described previously [33,34].

The study population consisted of workers with low SEP recruited from Danish workplaces within cleaning, transportation, manufacturing, construction, road maintenance, garbage disposal, assembly, mobile plant operator, and health care [33,34]. Eligible workers were employed in one of the mentioned sectors for at least 20 h/week; between 18–65 years old; and had given voluntary consent to participate. Workers were excluded if they were pregnant, had fever on the day of testing, or band-aid allergy.

The DPhacto and NOMAD studies were approved by the local Ethics Committee (file number H-2-2012-011 [33] and file number H-2-2011-047 [34], respectively). Both studies were conducted according to the Helsinki declaration and all data were anonymized in relation to individuals and workplaces.

### 2.1. Data Collection

Data were collected over four consecutive days and included questionnaires, health checks, and accelerometer-based measurements [33,34]. On day one, eligible workers were invited to complete a questionnaire and to participate in a health check, which consisted of anthropometric measurements and a physical health examination. Participants were asked to wear accelerometers for a minimum of two consecutive workdays and to complete a diary reporting time at work, time in bed at night and non-wear time.

### 2.2. Measurements

#### 2.2.1. Accelerometer Measurements of Physical Activity

Physical activity at work and leisure time was assessed using data from two tri-axial ActiGraph GT3X+ accelerometers (Actigraph, Pensacola, FL, USA). The accelerometers were fixed using double sided adhesive tape (3 M, Hair-Set, St. Paul, MN, USA) and Fixomull (Fixomull BSN medical GmbH, Hamburg, Germany) and placed on the upper back and right thigh. Accelerometer data were downloaded using Actilife Software version 5.5 (Actigraph, Pensacola, FL, USA) [35] and analyzed using the custom-made MATLAB program Acti4 (The National Research Centre for the Working Environment, Copenhagen, Denmark) [36]. The Acti4 program has been shown to separate physical activity types with high sensitivity and specificity under semi-standardized [36] and non-standardized conditions [37]. Classification of physical activity types using Acti4 has been described previously [36]. In brief, physical activity types (i.e., cycling, stair climbing, running, walking, standing, sitting and lying) were classified based on an algorithm using angles from the accelerometers axis and standard deviation of mean acceleration [36].

Daily work hours, leisure time and time in bed were defined from the participants’ self-reported diary information. Only workers with at least one day of valid accelerometer measurements of work and leisure time periods were included. A valid day consisted of ≥4 h of accelerometer-derived work and leisure time or ≥75% of the individual’s average work and leisure time. For workers with more than one valid day of accelerometer measurements, an average of daily time-use on occupational and leisure time physical activity was calculated. Average time in bed was based on accelerometer-derived periods of ≥4 h in bed at night.

Time-use of OPA and LTPA was treated as two subcompositions of activities performed within a 24 h day. OPA was defined as a 4-part subcomposition, consisting of time spent on walking, standing, sitting, and high intensity activities (HiPA: stair climbing, running and cycling). The parts of the OPA subcomposition were closed to a total time length of 480 min, corresponding to the workers’ average daily work hours. LTPA was defined as a 5-part subcomposition, consisting of time spent on sedentary behavior (sitting and lying), walking, standing, HiPA, and time in bed (TIB). The parts of the LTPA subcomposition were closed to a total time length of 960 min, equivalent to the workers’ average daily leisure time.

#### 2.2.2. Covariates

Sex and age of the workers were determined from each worker’s unique Danish civil registration number (CPR-number). BMI (Body Mass Index) was calculated as weight (kg) divided by height (m) squared (kg/m^2^). Information on shift work was assessed using the question: “At what time(s) of the day do you usually work in your main occupation?” with 3 response categories: fixed day work; night/varying work hours with night; and other. The variable was dichotomized into workers with fixed day work and workers with not fixed day work (including shift work and other). Information about pain in lower back, knees and feet/ankles was obtained by the questions: “Have you had any pain or trouble in the last 7 days in: lower back; knees; feet/ankles?” The answers were categorized into four categories for measurement of multi-site pain (no pain; pain in one body region; pain in two body regions; or pain in three body regions). Information on whether the worker was skilled was obtained by the question: “Are you skilled or unskilled?”. Missing data were not imputed.

### 2.3. Statistical Analysis

We used compositional data analysis to assess the association between OPA and LTPA. All analyses were performed using R Version 1.1.3 (RStudio, Boston, MA, USA) [38] using the “compositions” [39] and “robCompositions” packages [40].

Compositional mean, total variance, and variation matrix of the activities were calculated as descriptive data summaries [31,32]. In brief, the composition mean represents the central tendency for time-use of OPA and LTPA. Total variances were calculated for OPA and LTPA subcompositions from the respective sums of all log-ratio variances as a global measure of spread. Absolute and percentage contribution of each part to the total variance were calculated. The variation matrix measures co-dependence between activities in terms of proportionality with values close to 0 indicating that two parts are highly co-dependent.

#### 2.3.1. Ilr-Coordinates and Compositional Linear Regression Models

Following Chastin et al. and Hron et al., we used an isometric log-ratio (ilr) coordinate system to express the workers’ time-use for the OPA and LTPA subcompositions [30,41]. This system consisted of three and four ilr-coordinates for the OPA and LTPA subcompositions, respectively (the number of original parts, D, minus 1). Using this ilr representation, the first ilr-coordinate is given by the log-ratio of the part placed in the first position of the composition to the geometric mean of the remaining part within the composition. This way, all the information about the relative importance of that first part in the time-use distribution (with respect to the geometric average of the remaining parts) was contained in the first ilr-coordinate. The relative importance of the remaining parts of the OPA and LTPA subcompositions was subsequently isolated in the first ilr-coordinate by sequentially rearranging parts within the subcomposition, so that each part was put at the first position once. Hence, we obtained a number of alternative, although equivalent, ilr representations of the same subcomposition, which enabled investigation of associations between OPA and LTPA by regression analysis. In the following, we use the term relative importance to emphasize that time-use on one activity was assessed relative to time-use on the remaining activities.

Compositional linear regression models were used to estimate the direction and strength of the associations between activities performed at work and leisure time as represented by their relative importance in ilr-coordinates. In all models, the ilr-coordinates of the workers’ LTPA subcomposition acted as outcome variables, and the ilr-coordinates for the workers’ OPA subcomposition acted as exposure variables. This resulted in a total of 16 models fitted, each time rotating the set of ilr-coordinates used for outcome and exposure. See Appendix A (Table A1 and Table A2) of ilr-coordinate representation of the OPA and LTPA subcompositions and regression model fitting.

Potential confounders were chosen based on previous literature and theoretical assumptions concerning their possible influence on occupational and leisure time physical activity. The regression models were adjusted for the following covariates: age, BMI, shift work (not fixed day work vs. fixed day work (reference)), average work hours, and pain within the last 7 days in lower back and/or knees and/or feet/ankles (reference: no pain). Average work hours were calculated using the logarithm of the geometric mean of time spent on each part of the OPA subcomposition multiplied by √D = √4, as recommended in Pawlowsky-Glahn et al. [42]. Regression beta coefficients (($\beta_{1}$)) and standard errors were estimated for the 16 models. A MANOVA test on ilr-coordinates showed significant differences in the compositional mean of OPA and LTPA for men and women. Therefore, the regression models were stratified on sex. The assumptions of normality and homoscedasticity of the residuals were fulfilled for all models by visual inspection of plots of residuals versus predicted values and quantile-quantile plots. 

#### 2.3.2. Isotemporal Substitution Models

The first regression coefficients (($\beta_{1}$)) and 2-sided *p*-values for each model were used to determine if the relative importance of an occupational activity was statistically significantly associated with the relative importance of an activity during leisure time. *p*-values < 0.05 were considered statistically significant. Isotemporal substitution models were used to examine the effect size of reallocating time from one occupational activity to another, following the method proposed by Dumuid et al. [43]. HiPA was not considered for this analysis as less than 1% of the workers had values above 15 min of HiPA during work (data not shown). Consequently, results based on reallocating more than 15 min of work HiPA would not be reliable.

The isotemporal substitution analyses were conducted in multiple steps. Firstly, an expected LTPA composition was estimated based on the workers’ mean OPA composition. Secondly, new OPA compositions were made by reallocating time between the work activities (from 15 min to 60 min in 15-min increments). New LTPA subcompositions were estimated for each new OPA composition. Finally, expected changes in LTPA compositions were derived by taking the ilr-inverse of the LTPA ilr-coordinates estimated by the reference and new OPA compositions and then calculating change in leisure time activities. Thus, effect size was expressed as expected change in leisure time activities in minutes. An example of the procedure for obtaining change matrices is given in Appendix B.

#### 2.3.3. Sensitivity Analysis

It is possible that the effect of sex on the association between OPA and LTPA could be sector dependent. Therefore, sensitivity analyses were conducted in which only workers within manufacturing were included in a compositional linear regression analysis using same procedure as the primary analyses by adjusting for the same covariates and stratifying by sex.

## 3. Results

### 3.1. Study Population Characteristics

**Figure 1:**
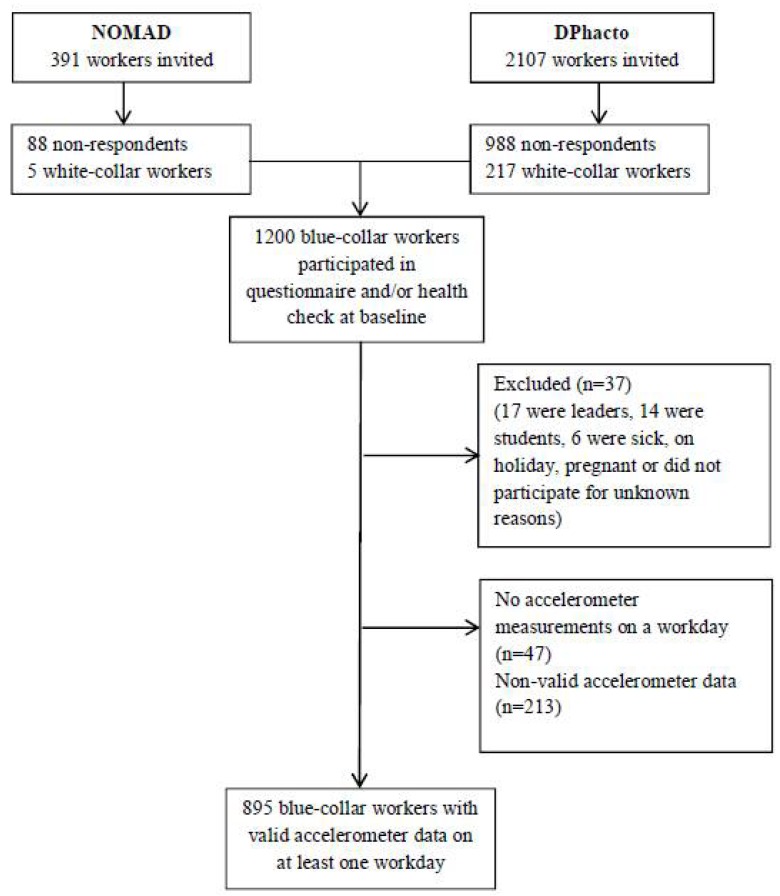
Flowchart of participants in the NOMAD and DPhacto study included in the current paper. NOMAD: New Method for Objective Measurements of Physical Activity in Daily Living; DPhacto: Danish PHysical ACTivity cohort with Objective measurements.

Figure 1 show the flowchart of workers included in the analyses. Initially, 391 and 2107 workers from the NOMAD and DPhacto studies were invited to participate, respectively. A total of 1200 eligible workers answered the baseline questionnaire and/or participated in the physical health check. Of these workers, 37 were excluded due to sickness on the day of testing; pregnancy; being students or department leaders; or for unknown reasons. Forty-seven workers were excluded from the analysis because they did not have accelerometer measurements on a workday and 213 workers were excluded due to non-valid accelerometer measurements. Therefore, a total of 895 workers were included in the analyses (495 men and 400 women).

### 3.2. Descriptive Statistics

Table 1 shows the baseline characteristics of the study population stratified by sex. Among men, the mean age was 46.6 (SD = 10.6); mean BMI was 27.1 (SD = 4.4); 74% had fixed day job; 32% were smokers; and the majority worked in manufacturing (67%). Among women, the mean age was 46.5 (SD = 8.8); mean BMI was 27.2 (SD = 0.7); 78% had a fixed day job; 34% were smokers; and most of the women worked in manufacturing (51%).

#### Compositional Descriptive Statistics

The compositional means showed that the majority of work time was spent standing for both men and women (Table 2). Most leisure time was spent in bed for both sexes, followed by sedentary behavior. There was a statistically significant difference between sexes in mean relative time spent on activities during work (*p* < 0.001) and leisure time (*p* < 0.001).

The compositional variation matrix in Table 3 shows the proportionality associations between work and leisure time activities. For example, for both men and women, standing and walking during work were the most associated activities (log-ratio variances τ = 0.23 and τ = 0.19, respectively). The contribution of each activity to the total variance of the subcompositions is shown in Table 3. For example, leisure time HiPA contributed to the highest variation for men (61%) and women (62%), suggesting that leisure time spent on HiPA varied substantially.

### 3.3. Primary Results

#### 3.3.1. Men

There was a statistically significant positive association between the relative importance of walking at work and walking at leisure time ($\hat{\beta }$ = 0.24, *p* < 0.01; Table 4) in men. Significant negative associations were observed between the relative importance of work time spent either standing or on HiPA and leisure time spent walking ($\hat{\beta }$ = −0.18, *p* < 0.01; and $\hat{\beta }$ = −0.08, *p* < 0.01; respectively).

The compositional isotemporal substitution analysis revealed that reallocating 15 min of work time to walking would be associated with an expected increase in leisure time walking of 2 min (Table 5). In contrast, increasing work time spent standing by 15 min would result in an expected decrease in leisure time walking of 1 min.

#### 3.3.2. Women

Among women, there was a statistically significant positive association between the relative importance of walking during work and the relative importance of sedentary behavior ($\hat{\beta }$ = 0.16, *p* = 0.03; Table 6) and walking ($\hat{\beta }$ = 0.12, *p* = 0.03) at leisure time (Table 6). The relative importance of time spent standing at leisure time was negatively associated with the relative importance of work time spent walking ($\hat{\beta }$ = −0.16, *p* = 0.01) and positively associated with the relative importance of work time spent standing ($\hat{\beta }$ = 0.15, *p* < 0.01). The relative importance of HiPA at work was significantly negatively associated with the relative importance of sedentary behavior ($\hat{\beta }$ = −0.12, *p* < 0.01) and positively associated with the relative importance of HiPA ($\hat{\beta }$ = 0.28, *p* < 0.01) during leisure time.

Results of the compositional isotemporal substitution analysis revealed that reallocating 15 min of work time spent walking would be associated with an expected increase in leisure time walking of 1 min; an expected increase in leisure time sedentary behavior of 4 min; and an expected decrease in leisure time standing of 7 min (Table 7).

### 3.4. Sensitivity Analysis

When only including workers within manufacturing in compositional regression analyses, the results were in accordance with those from the primary analysis. This indicates that the sex differences observed in associations between OPA and LTPA were not related to occupational sector differences in physical activity levels (results shown in Supplementary Tables S1 and S2).

## 4. Discussion

In this study, we investigated the association between OPA and LTPA among low SEP men and women. Overall, we found statistically significant associations between activities performed during work and leisure time for both sexes. However, the expected effect sizes were small. The largest effects were observed in women, for whom increasing work time walking by 15 min was associated with an increase in sedentary leisure time of 4 min and a decrease in leisure time standing of 7 min.

A 4 min and 7 min change in sedentary and standing leisure time, respectively, might appear small. However, these changes should be considered in relation to the women’s overall leisure time activities. The elevated risks of cardiovascular and all-cause mortality associated with sedentary behavior are most pronounced among inactive adults [44,45,46]. This population of women spent more than half of their waking leisure time being sedentary (~61%, Table 2). Consequently, the found combination of additional sedentary and reduced standing leisure time could have long-term health implications, potentially increasing the women’s mortality risk [47].

Extensive occupational walking can induce lower-extremity muscular fatigue and pain [15], which is likely to increase the workers’ need to compensate with an inactive leisure time. This was supported by the observed positive association between occupational walking and sedentary leisure time in women. Likewise, a study using accelerometer measurements from 445 workers reported that those performing more light intensity physical activity during work were less active during leisure time [21]. However, essential differences in study populations and methodology hinder direct comparison between the current study and Gay et al. [21]. 

For women, we found a statistically significant positive relationship between relative work and leisure time spent on HiPA (i.e., stair climbing, running and cycling, Table 6). This finding indicates a favorable association between high intensity OPA and LTPA in women. A possible explanation is that engaging in small amounts of high intensity physical activities throughout the workday does not generate the same level of fatigue as extensive light-intensity occupational activity, such as walking. Similarly, a study among 233 workers observed that for every 6 min additional moderate-to-vigorous physical activity (MVPA) during work, leisure time MVPA increased by 1 min [24]. However, the beneficial association between HiPA at work and leisure time is possibly a result of selection, with physically active workers being assigned to the most physically demanding work tasks. Unfortunately, our regression models showed a poor fit as less than 1% of the workers engaged in more than 15 min work HiPA (data not shown). Therefore, the findings regarding HiPA should be interpreted with caution.

We found no associations between relative time spent sitting at work and activities performed at leisure time for both sexes (Table 4 and Table 6). This did not match with our expectation that increasing occupational sitting in workers with manual jobs could be beneficial for fatigue and recovery, thereby facilitating an active leisure time. Similarly, two studies reported no difference in leisure time sitting or walking between blue-collar workers with high and low occupational sitting time [23,48]. Nevertheless, the potential of increasing occupational sitting to enhance recovery and energy for engaging in LTPA in workers with manual jobs needs to be investigated further.

Interestingly, we found dissimilar associations between OPA and LTPA in men and women. In men, OPA was only associated with relative leisure time walking (Table 4) whereas in women, OPA was associated with relative leisure time spent on sedentary behavior, walking and standing (Table 6). To assess if the sex differences in associations between OPA and LTPA were related to occupational sector, we conducted sensitivity analyses with only manufacturing workers included. These analyses showed results similar to those in our primary analyses, indicating that the discrepancies in associations between OPA and LTPA in men and women were not related to occupational sector (results shown in Supplementary Tables S1 and S2). Nevertheless, it is possible that the differences by sex in associations between OPA and LTPA are influenced by other factors related to occupation or biological dissimilarities [49,50,51]. Identifying potential mediators of the effect of OPA on LTPA is an important next step for determining factors which influence LTPA in low SEP men and women.

Men and women differed in mean work and leisure time activity profiles (Table 2). Men tended to spend more relative work time sitting compared with women. By contrast, men tended to spend less relative leisure time walking and standing compared with women. The differences in OPA could be related to men and women within the same job category performing different work tasks [52]. The dissimilarities in LTPA are possibly reflecting women performing more domestic work, such as cleaning and caring for children. This is in line with previous studies, reporting an unequal distribution of domestic tasks, with the majority being the women’s responsibility [53,54]. Hence, the pattern and determinants of OPA and LTPA in low SEP men and women is complex, possibly affected by a network of conditions and responsibilities at work and leisure.

### Strength and Limitations

A major methodological strength of this study was the use of a novel compositional data analysis approach. Using compositional regression models enabled assessment of the association between specific activities performed during work and leisure time, while accounting for the effects of other activities. Moreover, we obtained measurements of physical activities by means of accelerometer and a custom-made Acti4 program, with high sensitivity and specificity [36]. This study population showed physical activity patterns similar to other low SEP populations [6,55,56]. Accordingly, with some caution we consider our findings to be generalizable to other groups of low SEP workers within the same occupations. Finally, OPA and LTPA are closely related to SEP level [6]. Thus, our large study population of low SEP workers limits the possibility of socioeconomic confounding on the association between OPA and LTPA.

A limitation in this study was the low variation in LTPA, which could have attenuated the estimated associations between OPA and LTPA [57]. Accordingly, women in our dataset had a larger variation in both OPA and LTPA compared with men, which could explain that we found the largest effect sizes in women. Moreover, educational level is a potential confounder of the association between OPA and LTPA in this population of low SEP workers [6]. However, taken the distribution of skilled/unskilled workers into account did not alter the results (data not shown). Finally, this was a cross-sectional study and therefore the estimates and predictions found between OPA and LTPA should be interpreted with care. As with all cross-sectional studies, causal inference is limited and the estimated effect might reflect associations rather than actual causal effects.

## 5. Conclusions

In this study, we found weak relationships between OPA and LTPA in men. Among women, we found that reallocating 15 min of walking time at work was expected to increase sedentary leisure time by 4 min and decrease standing leisure time by 7 min. As this group of women engaged in little LTPA, any additional sedentary leisure time could have severe health implications. Our findings add new insight to the relationship between physical activities during work and leisure time. Future studies should investigate strategies for ensuring work conditions that facilitate a physically active leisure time in women with manual jobs, for example, breaking up long periods of work time on feet. Moreover, studies assessing potential moderating factors of the association between OPA and LTPA, such as age or physical capacity, are warranted for understanding factors which influence LTPA in low SEP groups.

## Figures and Tables

**Table 1:** Baseline characteristics of the study population, stratified on sex.

Variables	Men (N = 495)				Women (N = 400)			
	N	%	Mean (SD)	Range	N	%	Mean (SD)	Range
Age in years	495	100	46.6 (10.6)	[18.0; 68.0]	400	100	46.5 (8.8)	[21.0; 68.0]
Seniority in years	475	96	13.6 (10.8)	[0.0; 45.1]	376	94	12.5 (9.9)	[0.1; 48.0]
Overall health (1–5) ^A^	483	98	2.2 (0.7)	[1.0; 5.0]	393	98	2.3 (0.7)	[1.0; 5.0]
BMI in kg/m^2^	486	98	27.1 (4.4)	[18.7; 45.1]	394	99	27.2 (5.5)	[16.2; 43.8]
Aerobic capacity (mL O_2_/min/kg)	392	79	33.7 (9.1)	[13.9; 70.8]	276	69	29.8 (8.7)	[13.6; 8.9]
Alcohol consumption (units/week)	490	99	4.8 (6.1)	[0.0; 40.0]	394	99	1.6 (2.4)	[0.0; 18.0]
Accelerometer-derived measured days	495	100	2.6 (1.0)	[1.0; 5.0]	400	100	2.5 (0.9)	[1.0; 5.0]
Fixed day job	368	74			310	78		
Smokers	157	32			136	34		
Skilled workers	253	51			131	33		
Cohort								
NOMAD	125	25			95	24		
DPhacto	370	75			305	76		
Working sector								
Cleaning	20	4			142	35		
Manufacturing	330	67			204	51		
Transportation	58	12			2	1		
Health Service	0	0			17	4		
Assemblers	4	1			28	7		
Construction	38	8			0	0		
Garbage Collectors	21	4			0	0		
Mobile Plant Operators	11	2			0	0		
Other ^B^	13	2			7	2		
Multisite pain the last 7 days ^C^								
No pain	172	35			149	38		
Pain in one body region	203	41			148	37		
Pain in two body regions	80	16			79	20		
Pain in three body regions	37	8			21	5		

SD = standard deviation. BMI = body mass index. ^A^ High scores indicate high self-reported health. ^B^ Includes general office clerks and other elementary workers. ^C^ Body regions are lower back, knees and feet/ankles.

**Table 2:** Compositional mean for occupational physical activities and leisure time physical activities (in min/day and %).

Physical Activity	Men (N = 495)		Women (N = 400)		F-Test Statistic	*p*-Value
OPA (CM)						
	Min/day	%	Min/day	%		
Walking	87	18	87	18	18.901	<0.001
Standing	212	44	258	53		
Sitting	177	37	132	28		
HiPA	4	1	3	1		
LTPA (CM)						
	Min/day	%	Min/day	%		
SB	350	36	322	33	14.616	<0.001
Walking	42	4	50	5		
Standing	126	13	150	16		
HiPA	3	1	4	1		
TIB	439	46	434	45		

HiPA = high intensity physical activities (stair climbing, running and cycling). OPA = occupational physical activity. LTPA = leisure time physical activity. SB = sedentary behavior (sitting and lying). TIB = time in bed. Difference in OPA and LTPA subcomposition between sexes tested with MANOVA. Time-use of OPA was closed to the workers’ average daily work hours (480 min). Time-use of LTPA was closed to workers’ average daily leisure time (960 min).

**Table 3:** Compositional variation matrix for time spent on occupational physical activities and leisure time physical activities.

**Compositional Variation Matrix for OPA**												
**Men (N = 495)**							**Women (N = 400)**					
	Walking	Standing	Sitting	HiPA		Var-clr (%)	Walking	Standing	Sitting	HiPA		Var-clr (%)
Walking	0.00					0.35 (22%)	0.00					0.19 (12%)
Standing	0.23	0.00				0.13 (8%)	0.19	0.00				0.13 (9%)
Sitting	0.99	1.55	0.00			0.51 (33%)	1.19	1.24	0.00			0.69 (45%)
HiPA	0.65	1.23	1.35	0.00		0.57 (37%)	0.70	0.93	1.77	0.00		0.53 (34%)
Total var						1.56 (100%)						1.54 (100%)
**Compositional Variation Matrix for LTPA**												
**Men (N = 495)**							**Women (N = 400)**					
	SB	Walking	Standing	HiPA	TIB	Var-clr (%)	SB	Walking	Standing	HiPA	TIB	Var-clr (%)
SB	0.00					0.21 (13%)	0.00					0.19 (13%)
Walking	0.30	0.00				0.13 (8%)	0.25	0.00				0.11 (8%)
Standing	0.31	0.16	0.00			0.13 (8%)	0.29	0.12	0.00			0.11 (8%)
HiPA	1.56	1.21	1.25	0.00		1.01 (61%)	1.69	1.31	1.24	0.00		0.88 (62%)
TIB	0.12	0.23	0.23	1.52	0.00	0.16 (10%)	0.10	0.19	0.20	1.52	0.00	0.13 (9%)
Total var						1.64 (100%)						1.42 (100%)

HiPA = high intensity physical activities (stair climbing, running and cycling). OPA = occupational physical activity. LTPA = leisure time physical activity. SB = sedentary behavior (sitting and lying). TIB = time in bed. Total var = total variance of the subcomposition; Var-clr (%) = absolute and percentage (%) contribution of each part to the total variance. Values close to 0 indicate that two parts are nearly proportional (highly co-dependent) and thus, their log-ratio is nearly constant.

**Table 4:** Compositional analysis of the association between ilr-coordinates of OPA and LTPA subcompositions among men (N = 495).

OPA	LTPA											
	$\mathrm{ilr}(y_{1}$)α ln (SB: Walk, Stand, HiPA & TIB)			$\mathrm{ilr}(y_{1}$)α ln (Walk: Stand, HiPA, SB & TIB)			$\mathrm{ilr}(y_{1}$)α ln (Stand: HiPA, SB, Walk & TIB)			$\mathrm{ilr}(y_{1}$)α ln (HiPA: SB, Walk, Stand & TIB)		
	$\hat{\beta_{1}}$	SE	*p*-Value	$\hat{\beta_{1}}$	SE	*p*-Value	$\hat{\beta_{1}}$	SE	*p*-Value	$\hat{\beta_{1}}$	SE	*p*-Value
**ilr($z_{1}$)α** **ln (walk: stand, sit & HiPA)**	−0.06	0.06	0.33	0.24	0.05	<0.01	0.01	0.05	0.81	−0.05	0.15	0.72
**ilr($z_{1}$)α** **ln (stand: sit, walk & HiPA)**	0.03	0.05	0.49	−0.18	0.04	<0.01	−0.01	0.04	0.68	0.05	0.12	0.66
**ilr($z_{1}$)α** **ln (sit: walk, stand & HiPA)**	−0.01	0.04	0.75	0.02	0.03	0.58	0.02	0.03	0.56	−0.03	0.09	0.74
**ilr($z_{1}$)α** **ln (HiPA: walk, stand & sit)**	0.04	0.03	0.14	−0.08	0.02	<0.01	−0.01	0.02	0.53	0.03	0.07	0.61

HiPA = high intensity physical activities (stair climbing, running and cycling). OPA = occupational physical activity. LTPA = leisure time physical activity. SB = sedentary behavior (sitting and lying). SE = standard error. TIB = time in bed. $z_{1}$ = first ilr-coordinate of the OPA subcomposition. $y_{1}$ = first ilr-coordinate of the LTPA subcomposition. $\hat{\beta_{1}}$ = beta-coefficient associated to the first ilr-coordinate of the OPA subcomposition. Models adjusted for age, BMI, shiftwork, pain in back and/or knee and/or hip (multisite pain) and work hours.

**Table 5:** Expected difference in LTPA following reallocation between occupational physical activities among men (N = 495).

LTPA	SB		Walk		Stand		HiPA		TIB	
	min	Δ	min	Δ	min	Δ	min	Δ	min	Δ
Increasing Occupational Walking										
Original OPA composition	251		34		111		5		559	
+15 min walk	252	1	36 **	2	113	2	5	0	553	−4
+30 min walk	253	2	37 **	3	115	4	5	0	551	−8
+45 min walk	253	2	39 **	5	116	5	5	0	547	−12
+60 min walk	254	3	40 **	6	117	6	5	0	544	−15
Increasing Occupational Standing										
Original OPA composition	251		34		111		5		559	
+15 min stand	250	−1	33 **	−1	111	0	5	0	562	3
+30 min stand	250	−1	32 **	−2	110	−1	5	0	564	5
+45 min stand	249	−2	31 **	−3	109	−2	5	0	567	8
+60 min stand	248	−3	31 **	−3	108	−3	5	0	569	10
Increasing Occupational Sitting										
Original OPA composition	251		34		111		5		559	
+15 min sit	251	0	34	0	112	1	5	0	559	0
+30 min sit	250	−1	34	0	112	1	5	0	559	0
+45 min sit	250	−1	34	0	112	1	4	−1	560	1
+60 min sit	250	−1	34	0	112	1	4	−1	560	1

HiPA = high intensity physical activities (stair climbing, running and cycling). LTPA = leisure time physical activity. OPA = occupational physical activity. SB = sedentary behavior (sitting and lying). TIB = time in bed. Models adjusted for age, BMI, smoking-status, shiftwork, pain in back and/or knee and/or hip (multisite pain) and work hours. ** *p*-value < 0.01.

**Table 6:** Compositional analysis of the association between ilr-coordinates of OPA and LTPA subcompositions among women (N = 400).

OPA	LTPA											
	$\mathrm{ilr}(y_{1})$α ln (SB: Walk, Stand, HiPA & TIB)			$\mathrm{ilr}(y_{1})$α ln (Walk: Stand, HiPA, SB & TIB)			$\mathrm{ilr}(y_{1})$α ln (Stand: HiPA, SB, walk & TIB)			$\mathrm{ilr}(y_{1})$α ln (HiPA: SB, Walk, Stand & TIB)		
	$\hat{\beta_{1}}$	SE	*p*-Value	$\hat{\beta_{1}}$	SE	*p*-Value	$\hat{\beta_{1}}$	SE	*p*-Value	$\hat{\beta_{1}}$	SE	*p*-Value
**ilr($z_{1})$α** **ln (walk: stand, sit & HiPA)**	0.16	0.07	0.03	0.12	0.06	0.03	−0.16	0.06	0.01	−0.21	0.16	0.20
**ilr($z_{1})$α** **ln (stand: sit, walk & HiPA)**	−0.05	0.06	0.41	−0.04	0.06	0.52	0.15	0.05	<0.01	−0.03	0.14	0.85
**ilr($z_{1})$α** **ln(sit: walk, stand & HiPA)**	0.01	0.04	0.86	<0.01	0.03	0.92	0.02	0.03	0.39	−0.05	0.08	0.55
**ilr($z_{1})$α** **ln(HiPA: walk, stand & sit)**	−0.12	0.03	<0.01	−0.04	0.02	0.09	−0.01	0.02	0.59	0.28	0.07	<0.01

HiPA = high intensity physical activities (stair climbing, running and cycling). OPA = occupational physical activity. LTPA = leisure time physical activity. SB = sedentary behavior (sitting and lying). SE = standard error. TIB = time in bed. $z_{1}$ = first ilr-coordinate of the OPA subcomposition. $y_{1}$ = first ilr-coordinate of the LTPA subcomposition. $\hat{\beta_{1}}$ = beta-coefficient associated to the first ilr-coordinate of the OPA subcomposition. Models adjusted for age, BMI, shiftwork, pain in back and/or knee and/or hip (multisite pain) and work hours.

**Table 7:** Expected difference in LTPA following reallocation between occupational physical activities among women (N = 400).

LTPA	SB		Walk		Stand		HiPA		TIB	
	min	Δ	min	Δ	min	Δ	min	Δ	min	Δ
Increasing occupational walking										
Original OPA composition	215		63		218		13		450	
+15 min walk	219 *	4	64 *	1	211 *	−7	12	−1	453	3
+30 min walk	222 *	7	64 *	1	205 *	−13	12	0	456	6
+45 min walk	225 *	10	65 *	2	200 *	−18	12	0	458	8
+60 min walk	228 *	13	66 *	3	195 *	−23	11	−1	460	10
Increasing occupational standing										
Original OPA composition	215		63		218		13		450	
+15 min stand	214	−1	62	−1	221 **	3	13	0	450	0
+30 min stand	212	−3	62	−1	224 **	6	13	0	449	−1
+45 min stand	210	−5	61	−2	227 **	9	13	0	449	−1
+60 min stand	209	−6	61	−2	230 **	12	13	0	448	−2
Increasing occupational sitting										
Original OPA composition	215		63		218		13		450	
+15 min sit	215	0	63	0	219	1	13	0	451	1
+30 min sit	215	0	63	0	219	1	13	0	451	1
+45 min sit	215	0	63	0	219	1	13	0	451	1
+60 min sit	214	−1	63	0	219	1	13	0	451	1

HiPA = high intensity physical activities (stair climbing, running and cycling). LTPA = leisure time physical activity. OPA = occupational physical activity. SB = sedentary behavior (sitting and lying). TIB = time in bed. Models adjusted for age, BMI, smoking-status, shiftwork, pain in back and/or knee and/or hip (multisite pain) and work hours. * *p*-value < 0.05, ** *p*-value < 0.01.

## Supplementary Materials

The following are available online at http://www.mdpi.com/1660-4601/15/7/1306/s1, Table S1, Compositional linear regression analysis of the association between ilr-coordinates of occupational physical activity (OPA) and leisure time physical activity (LTPA) subcompositions among male manufacturers (N = 330), Table S2. Compositional linear regression analysis of the association between ilr-coordinates of occupational physical activity (OPA) and leisure time physical activity (LTPA) subcompositions among female manufacturers (N = 204).

## Appendix A

The associations between physical activities at work and leisure time were analyzed by multivariate linear regression models on isometric log-ratio (ilr) coordinates of the 4-part OPA and 5-part LTPA subcompositions of low socioeconomic workers.

Following [30,41], for a *D* − part composition $x=\left(x_{1},\ldots ,x_{D}\right)$ we can obtain a real vector $z=\left(z_{1},z_{2},\ldots ,\;z_{D-1}\right)$ of D−1 ilr-coordinates, where

(A1) $$z_{j=}\sqrt{\frac{D-j}{D-j+1}}\ln\frac{x_{j}}{\sqrt[{D-j}]{\prod_{k=j+1}^{D}x_{k}}},\;\;\;j=1,\ldots ,\;D-1$$

This is a particular choice of ilr-coordinates, recently called pivot coordinates [58], by which all relative information about the first part of the composition ($x_{1}$) is included in the first ilr-coordinate ($z_{1}$). The remaining ilr-coordinates ($z_{2},\;z_{3},\ldots ,\;z_{D}$) contain no information about the first part of the composition. This way, $z_{1}$ represents the relative importance or dominance of the part $x_{1}$ with respect to an (geometric) average of the remaining parts in the composition. Note that an infinite number of ilr coordinate systems can be defined; however, they are just geometric rotations from each other. This is a useful property in statistical analysis as it enables to use an arbitrary choice of ilr coordinates to obtain the required output.

Accordingly, with the objective of investigating the associations between parts of the OPA and LTPA subcompositions, the parts were sequentially rearranged (permuted) within the respective compositions to place each one of them at the first position before ilr transformation using Equation (A1).

For example, for the regression model 1a below, the ilr-coordinates of the 4-part OPA subcomposition were computed as

$$z_{1}^{*}=\sqrt{\frac{3}{4}}\ln\left(\frac{walk_{work_{i}}}{\sqrt[{3}]{stand_{work_{i}}*sit_{work_{i}}*HiPA_{work_{i}}}}\right)\;z_{2}^{*}=\sqrt{\frac{2}{3}}\ln\left(\frac{{stand}_{{work}_{i}}}{{\sqrt[{2}]{{sit}_{{work}_{i}}*HiPA_{work}}}_{i}}\right)\;z_{3}^{*}=\sqrt{\frac{1}{2}}\ln\left(\frac{{sit}_{{work}_{i}}}{{HiPA}_{{work}_{i}}}\right)$$

Giving rise to an OPA ilr-coordinate vector $ilr\left(Z\right)=\left(\begin{array}{c} z_{1}^{*}\\ z_{2}^{*}\\ z_{3}^{*} \end{array}\right)$.

The ilr-coordinates of the 5-part LTPA composition for model 1a were computed as

$$y_{1}^{*}=\sqrt{\frac{4}{5}}\ln\left(\frac{{SB}_{{leis}_{i}}}{\sqrt[{4}]{{walk}_{{leis}_{i}}*{stand}_{{leis}_{i}}*{HiPA}_{{leis}_{i}}*{TIB}_{i}}}\right)\;y_{2}^{*}=\sqrt{\frac{3}{4}}\ln\left(\frac{{walk}_{{leis}_{i}}}{\sqrt[{3}]{{stand}_{{leis}_{i}}*{HiPA}_{{leis}_{i}}*{TIB}_{i}}}\right)\;y_{3}^{*}=\sqrt{\frac{2}{3}}\ln\left(\frac{{stand}_{{leis}_{i}}}{{\sqrt[{2}]{{HiPA}_{{leis}_{i}}*{TIB}_{i}}}_{i}}\right)\;y_{4}^{*}=\sqrt{\frac{1}{2}}\ln\left(\frac{{HiPA}_{{leis}_{i}}}{{TIB}_{i}}\right)$$

Giving rise to a LTPA ilr-coordinate vector $ilr\left(Y\right)=\left(\begin{array}{c} y_{1}^{*}\\ y_{2}^{*}\\ y_{3}^{*}\\ y_{4}^{*} \end{array}\right)$.

The generic compositional linear regression model was then defined as

(A2) $$ilr\left(Y\right)=\beta_{0}+\beta^{\prime }ilr\left(Z\right)+covariates+\varepsilon =\beta_{0}+\beta_{1}z_{1}^{*}+\beta_{2}z_{2}^{*}+\beta_{3}z_{3}^{*}+covariates+\varepsilon$$

Like in ordinary regression, $\beta_{0}$ represents the model intercept; whereas $\beta_{1}$, $\beta_{2}$ and $\beta_{3}$ are the regression coefficients associated to each OPA ilr-coordinate in ilr(Z).

Based on Equation (A2), a total of 16 compositional linear regression models were constructed and fitted as described above, sequentially rearranging the first part of either the OPA or LTPA subcompositions (note that a model with dominance of LTPA time in bed, TIB, as response variable was not considered). Thus, permuting the OPA parts we defined models 1 to 4:

Model 1

$$\begin{array}{cc} ilr\left(Y\right)=\beta_{0}+ & \beta_{1}\sqrt{\frac{3}{4}}\ln\left(\frac{walk_{work_{i}}}{\sqrt[{3}]{stand_{work_{i}}*sit_{work_{i}}*HiPA_{work_{i}}}}\right)\\ & \;\;+\beta_{2}\sqrt{\frac{2}{3}}\ln\left(\frac{stand_{work}{}_{i}}{{\sqrt[{2}]{sit_{work}{}_{i}*HiPA_{work}}}_{i}}\right)+\beta_{3}\sqrt{\frac{1}{2}}\ln\left(\frac{sit_{work}{}_{i}}{HiPA_{work}{}_{i}}\right)+covariates\\ & \;\;+\varepsilon . \end{array}$$

Model 2

$$\begin{array}{cc} ilr\left(Y\right)=\beta_{0}+ & \beta_{1}\sqrt{\frac{3}{4}}\ln\left(\frac{stand_{work_{i}}}{\sqrt[{3}]{walk_{work_{i}}*sit_{work_{i}}*HiPA_{work_{i}}}}\right)\\ & \;\;+\beta_{2}\sqrt{\frac{2}{3}}\ln\left(\frac{walk_{work}{}_{i}}{{\sqrt[{2}]{sit_{work}{}_{i}*HiPA_{work}}}_{i}}\right)+\beta_{3}\sqrt{\frac{1}{2}}\ln\left(\frac{sit_{work}{}_{i}}{HiPA_{work}{}_{i}}\right)+covariates\\ & \;\;+\varepsilon \end{array}$$

Model 3

$$\begin{array}{cc} ilr\left(Y\right)=\beta_{0}+ & \beta_{1}\sqrt{\frac{3}{4}}\ln\left(\frac{sit_{work_{i}}}{\sqrt[{3}]{walk_{work_{i}}*stand_{work_{i}}*HiPA_{work_{i}}}}\right)\\ & \;\;+\beta_{2}\sqrt{\frac{2}{3}}\ln\left(\frac{walk_{work}{}_{i}}{{\sqrt[{2}]{stand_{work}{}_{i}*HiPA_{work}}}_{i}}\right)+\beta_{3}\sqrt{\frac{1}{2}}\ln\left(\frac{stand_{work}{}_{i}}{HiPA_{work}{}_{i}}\right)\\ & \;\;+covariates+\varepsilon \end{array}$$

Model 4

$$\begin{array}{cc} ilr\left(Y\right)=\beta_{0} & +\beta_{1}\sqrt{\frac{3}{4}}\ln\left(\frac{HiPA_{work}}{\sqrt[{3}]{walk_{work_{i}}*stand_{work_{i}}*sit_{work_{i}}}}\right)\\ & \;\;+\beta_{2}\sqrt{\frac{2}{3}}\ln\left(\frac{walk_{work}{}_{i}}{{\sqrt[{2}]{stand_{work}{}_{i}*sit_{work}}}_{i}}\right)+\beta_{3}\sqrt{\frac{1}{2}}\ln\left(\frac{stand_{work}{}_{i}}{sit_{work}{}_{i}}\right)\\ & \;\;+covariates+\varepsilon \end{array}$$

By then re-arranging LTPA parts for each model we obtained versions a, b, c and d:

1. Models 1a, 2a, 3a and 4a used $ilr\left(y_{1}^{*}\right)=\sqrt{\frac{4}{5}}\ln\left(\frac{SB_{leis}{}_{i}}{\sqrt[{4}]{walk_{leis}{}_{i}*stand_{leis}{}_{i}*HiPA_{leis}{}_{i}*TIB_{i}}}\right)$.
2. Models 1b, 2b, 3b and 4b used $ilr\left(y_{1}^{*}\right)=\sqrt{\frac{4}{5}}\ln\left(\frac{walk_{leis}{}_{i}}{\sqrt[{4}]{stand_{leis}{}_{i}*HiPA_{leis}{}_{i}*SB_{leis}{}_{i}*TIB_{i}}}\right)$.
3. Models 1c, 2c, 3c and 4c used $ilr\left(y_{1}^{*}\right)=\sqrt{\frac{4}{5}}\ln\left(\frac{stand_{leis}{}_{i}}{\sqrt[{4}]{HiPA_{leis}{}_{i}*SB_{leis}{}_{i}*walk_{leis}{}_{i}*TIB_{i}}}\right)$.
4. Models 1d, 2d, 3d and 4d used $ilr\left(y_{1}^{*}\right)=\sqrt{\frac{4}{5}}\ln\left(\frac{HiPA_{leis}{}_{i}}{\sqrt[{4}]{SB_{leis}{}_{i}*walk_{leis}{}_{i}*stand_{leis}{}_{i}*TIB_{i}}}\right)$.

For each model, the most relevant statistical significance test for our purposes was then performed on the $\beta_{1}$ coefficient of the regression models, which accounts for the association between the first ilr-coordinates of the OPA and LTPA subcompositions after accounting for all the other covariates. Importantly, although we focus on $\beta_{1}$, all the other ilr-coordinates must be included in the model to account for the intrinsic inter-dependences between parts of the respective compositions.

Below are examples of output tables for model 1a and model 2b among men and women, respectively. Note that the $\beta_{1}$ coefficient (highlighted in output tables) corresponds to the first ilr-coordinate and to the result shown in Table 4 and Table 5. The results of the remaining models (i.e., model 1c, 1d, 2a, 2b, 2c, 2d, etc.) are shown in Table 4 and Table 5.

**Table A1:** Output table for model 1a (among men, N = 400).

Variable	Estimates for $ilr\left(y_{1}^{*}\right)$		Estimates for $ilr\left(y_{2}^{*}\right)$		Estimates for $ilr\left(y_{3}^{*}\right)$		Estimates for $ilr\left(y_{4}^{*}\right)$	
	$\hat{\beta_{1}}$	*p*-Value	$\hat{\beta_{2}}$	*p*-Value	$\hat{\beta_{3}}$	*p*-Value	$\hat{\beta_{4}}$	*p*-Value
Intercept	1.207	<0.001	−0.105	0.650	1.138	<0.001	−3.339	<0.001
$ilr\left(z_{1}^{*}\right)$	**−0.063**	**0.332**	0.235	<0.001	0.079	0.292	0.053	0.677
$ilr\left(z_{2}^{*}\right)$	0.014	0.736	−0.107	0.002	−0.049	0.292	−0.002	0.766
$ilr\left(z_{3}^{*}\right)$	−0.033	0.271	0.052	0.049	0.032	0.365	−0.021	0.719
Average work hours	0.065	0.433	0.079	0.272	0.055	0.565	−0.243	0.133
Age	0.002	0.324	−0.003	0.106	−0.001	0.688	0.005	0.233
BMI	0.022	<0.001	0.011	0.016	0.009	0.159	−0.031	0.002
Shift work	0.002	0.978	0.013	0.785	−0.065	0.312	−0.105	0.334
One pain-site	0.099	0.337	−0.178	0.048	−0.116	0.324	0.023	0.907
Two pain-sites	0.050	0.589	−0.214	0.008	−0.128	0.225	0.096	0.590
Three pain-sites	−0.004	0.964	−0.239	0.005	−0.253	0.019	0.264	0.146

$ilr\left(z_{1}^{*}\right)$ = first ilr-coordinate of the OPA subcomposition. $ilr\left(y_{1}^{*}\right)$ = first ilr-coordinate of the LTPA subcomposition $\hat{\beta_{1}}$ = beta-coefficient associated to the first ilr-coordinate of the OPA subcomposition.

**Table A2:** Output for model 2b (among women, N = 400).

Variable	Estimates for $ilr\left(y_{1}^{*}\right)$		Estimates for $ilr\left(y_{2}^{*}\right)$		Estimates for $ilr\left(y_{3}^{*}\right)$		Estimates for $ilr\left(y_{4}^{*}\right)$	
	Estimates for $\hat{\beta_{1}}$	*p*-Value	$\hat{\beta_{2}}$	*p*-Value	$\hat{\beta_{3}}$	*p*-Value	$\hat{\beta_{4}}$	*p*-Value
Intercept	−1.014	<0.001	0.535	0.064	0.496	0.204	−1.826	0.002
$ilr\left(z_{1}^{*}\right)$	**−0.080**	**0.095**	0.136	0.017	−0.032	0.677	−0.022	0.847
$ilr\left(z_{2}^{*}\right)$	0.101	0.041	−0.097	0.096	0.161	0.041	−0.210	0.071
$ilr\left(z_{3}^{*}\right)$	0.022	0.267	0.029	0.224	0.098	0.002	−0.180	0.001
Average work hours	0.026	0.686	0.090	0.245	−0.112	0.284	0.149	0.332
Age	0.001	0.793	−0.002	0.482	0.005	0.163	0.001	0.919
BMI	0.013	<0.001	0.007	0.077	0.011	0.034	−0.014	0.079
Shift work	−0.016	0.725	−0.023	0.677	0.006	0.939	−0.078	0.479
One pain-site	0.106	0.233	−0.053	0.613	0.481	0.735	−0.003	0.990
Two pain-sites	0.124	0.141	−0.051	0.609	0.017	0.899	−0.013	0.949
Three pain-sites	0.087	0.301	−0.094	0.347	−0.019	0.883	0.036	0.954

$ilr\left(z_{1}^{*}\right)$ = first ilr-coordinate of the OPA subcomposition. $ilr\left(y_{1}^{*}\right)$ = first ilr-coordinate of the LTPA subcomposition $\hat{\beta_{1}}$ = beta-coefficient associated to the first ilr-coordinate of the OPA subcomposition.

## Appendix B

As outlined in the manuscript, compositional isotemporal substitution models were used to determine expected changes in workers’ LTPA subcomposition when reallocating fixed time durations to one part of the OPA subcomposition from the remaining parts. A detailed description of the method based on ilr linear regression can be found in Dumuid et al. [43,59].

In short, using a reference *D*-part composition $x=\left(x_{1},\ldots ,x_{D}\right)$ as starting point, we can consider the reallocation of a fixed duration of time, Δt, from one part of the composition to another. In our case, the reference composition was equal to the compositional geometric mean of the workers’ OPA subcomposition. It was closed to sum up to 1 and hence expressed in proportions to facilitate manipulations. We can then consider a relative increase in $x_{1}$ by a factor 1+r with $-1<r<\frac{1-x_{1}}{x_{1}}$.

To maintain a total sum of 1 when multiplying the first compositional part by the constant (1+r), the remaining parts were reduced using a factor (1−s), calculated as

(A3) $$s=r\frac{x_{1}}{1-x_{1}}$$

Modifying the parameters r and s we can use compositional linear regression models to estimate the expected LTPA subcomposition from a change in workers’ daily OPA subcomposition.

### Example: Expected Change in Leisure Time Sedentary Behavior when Increasing Occupational Walking

The compositional geometric mean of daily activities during work for men (closed to 480 min) was (walking = 87 min; standing = 212 min; sitting = 177 min; HiPA = 4 min).

After closing to 1, this corresponded to the set of proportions [0.181; 0.443; 0.368; 0.008].

As proportion walking during work was equal to 0.181, the complementary part of the OPA subcomposition was equal to 1 − 0.181. The time reallocated to the first part of the compositions must be expressed as proportion. Thus, to relatively increase occupational walking by 15 min, we compute

$$r=\frac{\frac{15}{480}}{x_{1}}=\frac{\frac{15}{480}}{0.181}=0.172$$

Using Formula (A3) we obtain

$$s=0.172\frac{0.181}{1-0.181}=0.038$$

Multiplying the first part of the OPA subcomposition (i.e., walking) by r=0.172 and the remaining OPA parts by s=0.038, we obtained a new OPA subcomposition. This corresponded to OPA time proportions [0.215; 0.431; 0.339; 0.014]. By closing it to 480 min, we obtained [walking = 102 min; standing = 204 min; sitting = 170 min; HiPA = 4 min].

Then, ilr-coordinates were calculated from this new OPA subcomposition including a 15-min relocation to occupational walking.

To investigate the effect on LTPA of this move from the reference to the new OPA subcomposition, we first estimated the LTPA subcomposition based on the reference OPA subcomposition using the following ilr multivariate regression model Equation (A3):

(A4) $$\begin{array}{c} ilr\left(Y\right)=\beta_{0}+\beta_{1}\sqrt{\frac{3}{4}}\ln\left(\frac{walk_{work_{i}}}{\sqrt[{3}]{stand_{work_{i}}*sit_{work_{i}}*HiPA_{work_{i}}}}\right)+\\ \beta_{2}\sqrt{\frac{2}{3}}\ln\left(\frac{stand_{work}{}_{i}}{{\sqrt[{2}]{sit_{work}{}_{i}*HiPA_{work}}}_{i}}\right)+\beta_{3}\sqrt{\frac{1}{2}}\ln\left(\frac{sit_{work}{}_{i}}{HiPA_{work}{}_{i}}\right)+covariates+\varepsilon \end{array}$$

where the LTPA ilr-coordinate vector $ilr\left(Y\right)=\left(\begin{array}{c} y_{1}^{*}\\ y_{2}^{*}\\ y_{3}^{*}\\ y_{4}^{*} \end{array}\right)$ consisted of

$$y_{1}^{*}=\sqrt{\frac{4}{5}}\ln\left(\frac{SB_{leis}{}_{i}}{\sqrt[{4}]{walk_{leis}{}_{i}*stand_{leis}{}_{i}*HiPA_{leis}{}_{i}*TIB_{i}}}\right)\;y_{2}^{*}=\sqrt{\frac{3}{4}}\ln\left(\frac{walk_{leis}{}_{i}}{\sqrt[{3}]{stand_{leis}{}_{i}*HiPA_{leis}{}_{i}*TIB_{i}}}\right)\;y_{3}^{*}=\sqrt{\frac{2}{3}}\ln\left(\frac{stand_{leis}{}_{i}}{{\sqrt[{2}]{HiPA_{leis}{}_{i}*TIB_{i}}}_{i}}\right)\;y_{4}^{*}=\sqrt{\frac{1}{2}}\ln\left(\frac{HiPA_{leis}{}_{i}}{TIB_{i}}\right)$$

The following beta coefficients matrix (**B**) was obtained from model Equation (A3):

$$B=\left[\begin{array}{cccc} 1.25 & -0.05 & 1.19 & -3.48\\ -0.02 & 0.19 & 0.07 & 0.02\\ 0.01 & -0.11 & -0.04 & -0.03\\ -0.01 & 0.04 & 0.03 & -0.05 \end{array}\right]$$

Using **B** in combination with the ilr-coordinates of the reference OPA subcomposition, we obtained expected ilr-coordinates of the LTPA subcomposition ($\hat{ilr\left(Y\right)}$ as:

$$\begin{array}{cc} \hat{ilr\left(y_{1}^{*}\right)}=1.25- & 0.02*\sqrt{\frac{3}{4}}\ln\left(\frac{walk_{work_{i}}}{\sqrt[{3}]{stand_{work_{i}}*sit_{work_{i}}*HiPA_{work_{i}}}}\right)+0.01\\ & *\sqrt{\frac{2}{3}}\ln\left(\frac{stand_{work}{}_{i}}{\sqrt[{2}]{sit_{work}{}_{i}*HiPA_{work_{i}}}}\right)-0.01*\sqrt{\frac{1}{2}}\ln\left(\frac{sit_{work}{}_{i}}{HiPA_{work}{}_{i}}\right)\\ & +covariates\\ \hat{ilr\left(y_{2}^{*}\right)}=-0.05+ & 0.19*\left(\frac{walk_{work_{i}}}{\sqrt[{3}]{stand_{work_{i}}*sit_{work_{i}}*HiPA_{work_{i}}}}\right)-0.11\\ & *\sqrt{\frac{2}{3}}\ln\left(\frac{stand_{work}{}_{i}}{\sqrt[{2}]{sit_{work}{}_{i}*HiPA_{work_{i}}}}\right)+0.04*\sqrt{\frac{1}{2}}\ln\left(\frac{sit_{work}{}_{i}}{HiPA_{work}{}_{i}}\right)\\ & +covariates\\ \hat{ilr\left(y_{3}^{*}\right)}=1.19+ & 0.07*\sqrt{\frac{3}{4}}\ln\left(\frac{walk_{work_{i}}}{\sqrt[{3}]{stand_{work_{i}}*sit_{work_{i}}*HiPA_{work_{i}}}}\right)-0.04\\ & *\sqrt{\frac{2}{3}}\ln\left(\frac{stand_{work}{}_{i}}{{\sqrt[{2}]{sit_{work}{}_{i}*HiPA_{work}}}_{i}}\right)+0.03*\sqrt{\frac{1}{2}}\ln\left(\frac{sit_{work}{}_{i}}{HiPA_{work}{}_{i}}\right)\\ & +covariates\\ \hat{ilr\left(y_{4}^{*}\right)}=-3.48+ & 0.02*\sqrt{\frac{3}{4}}\ln\left(\frac{walk_{work_{i}}}{\sqrt[{3}]{stand_{work_{i}}*sit_{work_{i}}*HiPA_{work_{i}}}}\right)-0.03\\ & *\sqrt{\frac{2}{3}}\ln\left(\frac{stand_{work}{}_{i}}{{\sqrt[{2}]{sit_{work}{}_{i}*HiPA_{work}}}_{i}}\right)-0.05*\sqrt{\frac{1}{2}}\ln\left(\frac{sit_{work}{}_{i}}{HiPA_{work}{}_{i}}\right)\\ & +covariates \end{array}$$

Resulting in the following expected LTPA ilr-coordinates:

$$\hat{ilr\left(Y\right)}=\left(\begin{array}{c} 1.32\\ -0.16\\ 1.03\\ -3.76 \end{array}\right)$$

This vector was transformed back by ilr-inverse transformation to express it as a composition [0.25; 0.05; 0.14; 0.003; 0.560]. By closing it to 960 min as originally, we obtained the expected LTPA subcomposition [SB 237 min; walking 50 min; standing 133 min; HiPA 3 min; TIB 538 min].

The same procedure was performed, but now using **B** in combination with the ilr-coordinates of the new OPA subcomposition after 15-min walking relocation, to obtain the corresponding expected LTPA composition for this case:

$$\begin{array}{l} \hat{ilr\left(y_{1}^{*}\right)=}1.25-0.02\\ {}*\sqrt{\frac{3}{4}}\ln\left(\frac{walk_{work_{i}}*\left(1+r\right)}{\sqrt[{3}]{stand_{work_{i}}*\left(1-s\right)*sit_{work_{i}}*\left(1-s\right)*HiPA_{work_{i}}*\left(1-s\right)}}\right)+0.01\\ {}*\sqrt{\frac{2}{3}}\ln\left(\frac{stand_{work}{}_{i}*\left(1-s\right)}{\sqrt[{2}]{sit_{work}{}_{i}*\left(1-s\right)*HiPA_{work_{i}}*\left(1-s\right)}}\right)-0.01*\sqrt{\frac{1}{2}}\ln\left(\frac{sit_{work}{}_{i}*\left(1-s\right)}{HiPA_{work}{}_{i}*\left(1-s\right)}\right)\\ +covariates\\ \hat{ilr\left(y_{2}^{*}\right)}=-0.05+0.19*\left(\frac{walk_{work_{i}}*\left(1+r\right)}{\sqrt[{3}]{stand_{work_{i}}*\left(1-s\right)*sit_{work_{i}}*\left(1-s\right)*HiPA_{work_{i}}*\left(1-s\right)}}\right)\\ \;-0.11*\sqrt{\frac{2}{3}}\ln\left(\frac{stand_{work}{}_{i}*\left(1-s\right)}{\sqrt[{2}]{sit_{work}{}_{i}*\left(1-s\right)*HiPA_{work_{i}}*\left(1-s\right)}}\right)+0.04\\ \;*\sqrt{\frac{1}{2}}\ln\left(\frac{sit_{work}{}_{i}*\left(1-s\right)}{HiPA_{work}{}_{i}*\left(1-s\right)}\right)+covariates\\ \hat{ilr\left(y_{3}^{*}\right)}\\ =1.19+0.07*\sqrt{\frac{3}{4}}\ln\left(\frac{walk_{work_{i}}*\left(1+r\right)}{\sqrt[{3}]{stand_{work_{i}}*\left(1-s\right)*sit_{work_{i}}*\left(1-s\right)*HiPA_{work_{i}}*\left(1-s\right)}}\right)\\ -0.04*\sqrt{\frac{2}{3}}\ln\left(\frac{stand_{work}{}_{i}*\left(1-s\right)}{\sqrt[{2}]{sit_{work}{}_{i}*\left(1-s\right)*HiPA_{work_{i}}*\left(1-s\right)}}\right)+0.03\\ {}*\sqrt{\frac{1}{2}}\ln\left(\frac{sit_{work}{}_{i}*\left(1-s\right)}{HiPA_{work}{}_{i}*\left(1-s\right)}\right)+covariates\\ \hat{ilr\left(y_{4}^{*}\right)}\\ =-3.48+0.02*\sqrt{\frac{3}{4}}\ln\left(\frac{walk_{work_{i}}*\left(1+r\right)}{\sqrt[{3}]{stand_{work_{i}}*\left(1-s\right)*sit_{work_{i}}*\left(1-s\right)*HiPA_{work_{i}}*\left(1-s\right)}}\right)\\ -0.03*\sqrt{\frac{2}{3}}\ln\left(\frac{stand_{work}{}_{i}*\left(1-s\right)}{\sqrt[{2}]{sit_{work}{}_{i}*\left(1-s\right)*HiPA_{work_{i}}*\left(1-s\right)}}\right)-0.05\\ {}*\sqrt{\frac{1}{2}}\ln\left(\frac{sit_{work}{}_{i}*\left(1-s\right)}{HiPA_{work}{}_{i}*\left(1-s\right)}\right)+covariates \end{array}$$

Using the parameters r=0.172 and s=0.038, the following expected LTPA ilr-coordinates and subcomposition (in proportions) were obtained:

$$\hat{ilr\left(Y\right)}=\left(\begin{array}{c} 1.92\\ -0.10\\ 1.04\\ -3.75 \end{array}\right)$$

and [0.25; 0.05; 0.14; 0.003; 0.555], respectively. After closing to 960 min, the expected LTPA subcomposition was [SB 238 min; walking 52 min; standing 135 min; HiPA 3 min; TIB 533 min].

By taking the change in $\hat{ilr\left(Y\right)}$ predicted by the reference and new OPA subcomposition, it was possible to estimate the expected change in leisure time activities. The same procedure was performed for each new OPA subcomposition where time had been reallocated. We expressed the change matrices as minutes changed in leisure time activities by taking the inverse ilr transformation of the model predictions (see Table 5 and Table 7 for results).
