# Supplementary material for: Does Physically Demanding Work Hinder a Physically Active Lifestyle in Low Socioeconomic Workers? A Compositional Data Analysis Based on Accelerometer Data
Source: Int J Environ Res Public Health. 2018 Jun 21;15(7):1306. doi: 10.3390/ijerph15071306 (PMC6068990; doi:10.3390/ijerph15071306)
Supplement: Supplementary file 1 [file ijerph-15-01306-s001.pdf]

Table S1. Compositional linear regression analysis of the association between ilr-coordinates of occupational physical activity (OPA) and leisure time physical activity (LTPA) subcompositions among male manufacturers (N = 330).

	LTPA											
	ilr(y_1)∝ ln(SB : walk, stand, HiPA & TIB)			ilr(y_1)∝ ln(walk : stand, HiPA, SB & TIB)			ilr(y_1)∝ ln(stand : HiPA, SB, walk & TIB)			ilr(y_1)∝ ln(HiPA: SB, walk , stand & TIB)		
**OPA**	$\widehat{\beta_1}$	SE	*p*-value	$\widehat{\beta_1}$	SE	*p*-value	$\widehat{\beta_1}$	SE	*p*-value	$\widehat{\beta_1}$	SE	*p*-value
**ilr(z_1)∝ ln (walk: stand, sit & HiPA)**	0.15	0.09	0.10	0.22	0.07	<0.01	−0.01	0.08	0.85	−0.53	0.21	0.01
**ilr(z_1)∝ ln (stand: sit, walk & HiPA)**	−0.11	0.07	0.12	−0.17	0.06	<0.01	0.03	0.06	0.59	0.40	0.16	0.01
**ilr(z_1)∝ ln(sit: walk, stand & HiPA)**	−0.04	0.04	0.32	0.02	0.04	0.64	0.01	0.04	0.81	0.06	0.10	0.53
**ilr(z_1)∝ ln(HiPA: walk, stand & sit)**	0.15	0.09	0.10	0.22	0.07	<0.01	−0.01	0.08	0.85	−0.53	0.21	0.01

HiPA= high intensity physical activities (stair climbing, running and cycling); SB = sedentary behaviour (sitting and lying); SE = standard error; TIB = time in bed; z_1= first ilr-coordinate of the OPA subcomposition; y_1= first ilr-coordinate of the LTPA subcomposition; $\widehat{\beta_1}$= beta-coefficient associated to the first ilr-coordinate of the OPA subcomposition. Models adjusted for age, BMI, shiftwork, pain in back and/or knee and/or hip (multisite pain) and work hours.

Table S2. Compositional linear regression analysis of the association between ilr-coordinates of occupational physical activity (OPA) and leisure time physical activity (LTPA) subcompositions among female manufacturers (N = 204).

	LTPA											
	ilr(y_1)∝ ln(SB : walk, stand, HiPA & TIB)			ilr(y_1)∝ ln(walk : stand, HiPA, SB & TIB)			ilr(y_1)∝ ln(stand : HiPA, SB, walk & TIB)			ilr(y_1)∝ ln(HiPA: SB, walk , stand & TIB)		
**OPA**	$\widehat{\beta_1}$	SE	*p*-value	$\widehat{\beta_1}$	SE	*p*-value	$\widehat{\beta_1}$	SE	*p*-value	$\widehat{\beta_1}$	SE	*p*-value
**ilr(z_1)∝ ln (walk: stand, sit & HiPA)**	0.22	0.09	0.02	0.11	0.07	0.11	−0.13	0.07	0.07	−0.37	0.19	0.06
**ilr(z_1)∝ ln (stand: sit, walk & HiPA)**	−0.18	0.08	0.03	−0.07	0.06	0.26	0.12	0.06	0.05	0.27	0.17	0.12
**ilr(z_1)∝ ln(sit: walk, stand & HiPA)**	−0.01	0.04	0.80	<0.01	0.03	0.89	0.03	0.02	0.36	−0.03	0.09	0.77
**ilr(z_1)∝ ln(HiPA: walk, stand & sit)**	0.22	0.09	0.02	0.11	0.07	0.11	-0.13	0.07	0.07	−0.37	0.19	0.06

HiPA= high intensity physical activities (stair climbing, running and cycling); SB=sedentary behaviour (sitting and lying); SE=standard error; TIB = time in bed; z_1= first ilr-coordinate of the OPA subcomposition; y_1= first ilr-coordinate of the LTPA subcomposition; $\widehat{\beta_1}$= beta-coefficient associated to the first ilr-coordinate of the OPA subcomposition. Models adjusted for age, BMI, shiftwork, pain in back and/or knee and/or hip (multisite pain) and work hours.
